# Regulation of Apoptotic Effects by Erythrocarpine E, a Cytotoxic Limonoid from *Chisocheton erythrocarpus* in HSC-4 Human Oral Cancer Cells

**DOI:** 10.1371/journal.pone.0023661

**Published:** 2011-08-17

**Authors:** Noor Hasima Nagoor, Norliza Shah Jehan Muttiah, Chong Soon Lim, Lionel L. A. In, Khalit Mohammad, Khalijah Awang

**Affiliations:** 1 Faculty of Science, Institute of Biological Science (Genetics and Molecular Biology), University Malaya, Kuala Lumpur, Malaysia; 2 Department of Chemistry, Faculty of Science, Centre for Natural Product Research and Drug Discovery (CENAR), University Malaya, Kuala Lumpur, Malaysia; 3 Department of Pharmacy, Faculty of Medicine, University Malaya, Kuala Lumpur, Malaysia; MRC National Institute for Medical Research, United States of America

## Abstract

The aim of this study was to determine the cytotoxic and apoptotic effects of erythrocarpine E (CEB4), a limonoid extracted from *Chisocheton erythrocarpus* on human oral squamous cell carcinoma. Based on preliminary dimethyl-2-thiazolyl-2,5-diphenyl-2H-tetrazolium bromide (MTT) assays, CEB4 treated HSC-4 cells demonstrated a cytotoxic effect and inhibited cell proliferation in a time and dose dependent manner with an IC_50_ value of 4.0±1.9 µM within 24 h of treatment. CEB4 was also found to have minimal cytotoxic effects on the normal cell line, NHBE with cell viability levels maintained above 80% upon treatment. Annexin V-fluorescein isothiocyanate (FITC), poly-ADP ribose polymerase (PARP) cleavage and DNA fragmentation assay results showed that CEB4 induces apoptosis mediated cell death. Western blotting results demonstrated that the induction of apoptosis by CEB4 appeared to be mediated through regulation of the p53 signalling pathway as there was an increase in p53 phosphorylation levels. CEB4 was also found to up-regulate the pro-apoptotic protein, Bax, while down-regulating the anti-apoptotic protein, Bcl-2, suggesting the involvement of the intrinsic mitochondrial pathway. Reduced levels of initiator procaspase-9 and executioner caspase-3 zymogen were also observed following CEB4 exposure, hence indicating the involvement of cytochrome c mediated apoptosis. These results demonstrate the cytotoxic and apoptotic ability of erythrocarpine E, and suggest its potential development as a cancer chemopreventive agent.

## Introduction

Various natural phytocompounds have been screened and investigated extensively as potential anti-cancer agents. Approximately 74% of drugs that were approved for cancer treatment have been extracted from natural sources, either created by structural modification or synthesized and designed based on natural compounds as a model [1]. Most of natural anti-cancer agents available to date are derived from plants, animals, marine organisms and microorganisms. Some currently used examples of plant-derived compounds with potential anti-cancer activities are 1′S-1′acetoxyeugenol acetate and 1′S-1′-acetoxychavicol acetate [2], vincristine, irinotecan, etoposide and paclitaxel, while bleomycin and doxorubicin are microbial-derived compounds, and citarabine, derived from marine sources [3].

A subgroup of natural compounds known as limonoids, are highly oxygenated tetracyclic triterpene derivatives and have been recognized to possess interesting biological activities. To date, many limonoids have been isolated with a wide range of biological implications including insect anti-feedant, growth inhibiting characteristics and anti-inflammatory agents [4]. Several citrus limonoid aglycones such as limonin, nomilin, obacunone, isoobacunoic acid and ichangin have recently been subjected to anti-cancer screening procedures, and were found to induce significant glutathione-S-transferase (GST) and quinine reductase (QR) activity in the liver and intestinal mucosa of mice and rats respectively [5]–[6]. Other reports include indications that mixtures of limonoid aglycones with limonoid glucosides were equipotent to tamoxifen for inhibiting the proliferation of ER positive breast cancer cells, and an even higher potency against estrogen-independent breast cancer cells [7]. Limonoids from the ethanolic extract of *Azadirachta indica* (neem bark, leaves and seed oil) were also found to cause cell death of prostate cancer cells (PC-3) by inducing apoptosis through a reduction in Bcl-2 expression levels corresponding with an increase in Bax levels [8].

Currently, the process of apoptosis and its relevance in cancer as the preferred mode of death due to the effective clearance of apoptotic cells without triggering inflammation has become one of the priority fields in cancer research [9]. Recent studies conducted on limonoids extracted from *Citrus aurantifolia* showed that the occurrence of apoptosis was mediated through the induction of caspase-3, driven by the intrinsic pathway and the release of cytochrome-c in human pancreatic cancer cells [10]. The natural limonoid, erythrocarpine E (CEB4) which was utilized in this study, is an A, B, D-seco heptacyclic limonoid with a cinnamoyl group as the side chain at C-3 extracted from the bark of *Chisocheton erythrocarpus* Hiern from the Meliaceae family, commonly known as ‘Rongga’ in Malaysia. It has recently been demonstrated that CEB4 induces cytotoxic activity against P-388 murine leukemia cells at IC_50_ of 16.0 µg/ml [11]. In this study, CEB4 was investigated for its potential to induce apoptosis in HSC-4 human oral squamous cell carcinoma (SCC).

## Materials and Methods

### Plant material

*Chisocheton erythrocarpus* Hiern was collected from Hutan Simpan Terenas, Kedah, Malaysia. The sample was identified by Mr. Teoh Leng Eng from the Dept. of Chemistry, Faculty of Science, University of Malaya. A voucher specimen (KL 4863) was deposited in the Department of Chemistry Herbarium, University of Malaya.

### Reagents

Dulbecco's modified Eagle's medium (DMEM) and Roswell Park Memorial Institute-1640 (RPMI-1640) with 4.5 g glucose/L, 300 mg/l L-glutamine, 2.5% (v/v) trypsin in modified Hank's balanced salt solution (HBSS) without calcium or magnesium, fetal bovine serum (FBS) and all antibiotics were purchased from Lonza Inc., USA. Dimethyl-2-thiazolyl-2,5-diphenyl-2H-tetrazolium bromide (MTT) reagent, annexin V-fluorescein isothiocyanate (FITC) apoptosis detection kit, propidium iodide (PI), RNase and SuicideTrack™ DNA Ladder Isolation kit were purchased from Calbiochem (CA, USA).

### Extraction and Isolation Natural Compound

Dried ground bark (1.3 kg) of *Chisocheton erythrocarpus* were extracted successively with hexane, dichloromethane and methanol. Each extract was then dried *in vacuo*. The dried dichloromethane extract (4.0 g) was subjected to fractionation by silica gel column chromatography using a solvent mixture of hexane-ethyl acetate. The polarity of the mobile phase was increased gradually to 100% ethyl acetate. The fraction eluted from 30% of ethyl acetate, which contained the target compound, was separated by preparative thin layer chromatography (TLC) with hexane-ethyl acetate (7:3) to yield erythrocarpine E (4.7 mg).

### Cell Lines and Culture Conditions

A set of five human tumor cell lines were used in this study, comprising HSC-4, HSC-2 oral tumor cell lines and Ca Ski cervical tumor cell line obtained from the Cancer Research Initiative Foundation (CARIF, Malaysia), MCF-7 breast adenocarcinoma and HepG2 hepatocarcinoma which were obtained from the University of Malaya Medical Center (UMMC), and normal human bronchial epithelial cells (NHBE) (Lonza Inc., USA) used as a normal cell control. For routine maintenance HSC-4, HSC-2, Ca Ski and HepG2 cells were cultured in DMEM and MCF-7 cells were cultured in RPMI-1640, with both media types supplemented with 10% (v/v) FBS, 100 U/ml penicillin and 100 µg/ml streptomycin. NHBE cells were cultured with bronchial epithelial basal medium (BEBM) with 10% (v/v) FBS, 100 U/ml penicillin and 100 µg/ml streptomycin. Cells were grown as monolayers at 37°C in humidified atmosphere with 5% CO_2_/95% air.

### MTT Cell Viability Assay

The cytotoxic effects of CEB4 on all cell lines were determined using the MTT assay method. CEB4 was dissolved in dimethyl sulfoxide (DMSO) to a final concentration of 10 mM. Briefly 1.0×10^4^ cells per 100 µl of media were seeded in each well of a 96-well plate in the presence or absence of CEB4 at final concentrations of 5 µM to 40 µM for up to 24 h. MTT (5 mg/ml) was added to each well (10 µl/well) and plates were incubated at 37°C for 2 h. After incubation, the medium was replaced with 200 µl DMSO and the absorbance was measured at 570 nm for each well using a microplate reader (Tecan Sunrise®, Switzerland).

### Annexin V-FITC/PI Analysis

Detection of apoptosis was conducted using the Annexin V-FITC/PI apoptosis detection kit according to manufacturer's protocol. Briefly, both CEB4 treated and untreated HSC-4 and NHBE cells were harvested by trypsinization, washed in 1x PBS and stained with annexin V-FITC conjugate and PI. Cells were then analyzed by flow cytometry (BD FACSCalibur™, USA) using BD CellQuest acquisition and analysis software.

### DNA Fragmentation Assay

Total DNA was extracted from both untreated and treated cells with CEB4 for 12 and 24 h using the SuicideTrack™ DNA Isolation Kit according to manufacturer's protocol. Isolated DNA was analysed on a 1% (w/v) agarose gel electrophoresis and stained with ethidium bromide. Fragmentation of DNA was observed under UV illumination using a gel documentation system (Alpha Inotech, USA).

### Western Blotting

CEB4 treated HSC-4 cells were grown to a cell density of 80–90% confluency, washed with ice-cold 1x PBS and proteins extracted using the NE-PER® Nuclear and Cytoplasmic Extraction Kit (Pierce, USA). Protein concentrations were determined using the Bio-Rad DC protein assay (Bio-Rad Laboratories, USA). Equal amounts of protein were subjected to 12% SDS-PAGE and transferred to a nitrocellulose membrane. The membranes were subsequently incubated with nine primary antibodies against β-actin, poly-ADP ribose polymerase (PARP), p53, phospho-p53, murine double minute 2 (MDM2), Bax, Bcl-2, procaspase-3 and procaspase-9. After washing in Tris-buffered saline and Tween-20 (TBST) buffer, horseradish peroxidase (HRP)-linked secondary antibodies were added and bound proteins were detected through enhanced chemiluminescence signals using x-ray films. Relative intensities of all bands were quantified using image analysis software (NIH ImageJ v1.43, National Institutes of Health, USA).

### Statistical Analysis

All results were expressed as mean ±s.d. of data obtained from three independent experiments. The statistical significance between various groups was determined using one-way ANOVA. Significant differences were considered as p≤0.05.

## Results

### Isolation and Characterization of Erythrocarpine E (CEB4)

**Figure 1:**
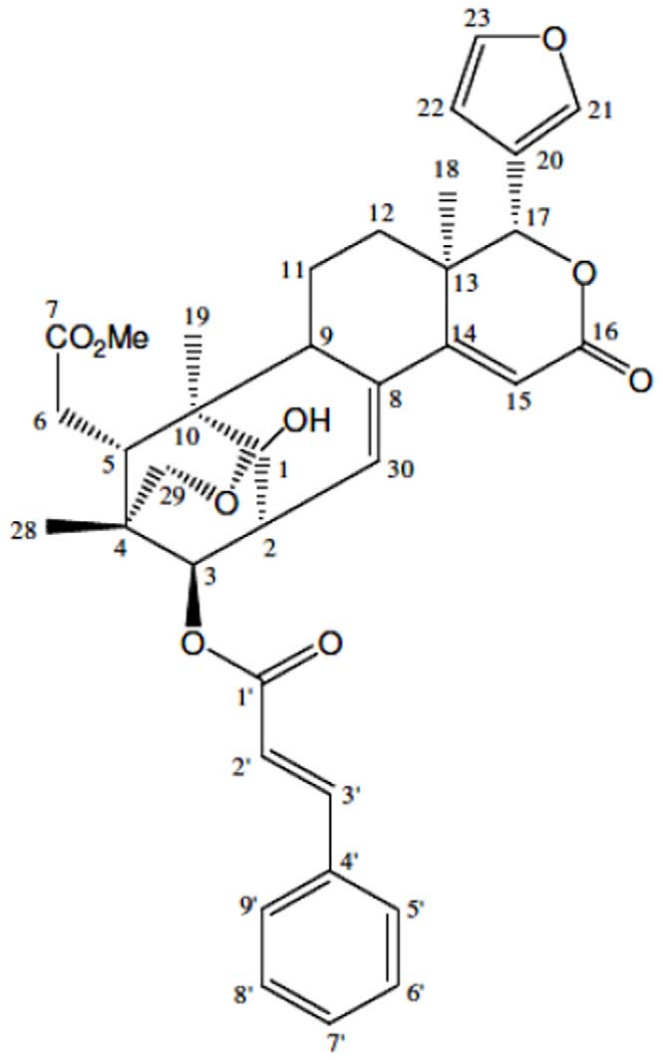
Chemical structure of erythrocarpine E (CEB4) extracted from *Chisocheton erythrocarpus.*

CEB4 (erythrocarpin E) was isolated as white amorphous powder. The molecular formula was C_36_H_40_O_10_, which was determined from the [M+H]^+^ peak at m/z 633.2703 in the high resolution fast atom bombardment mass spectrometry (HRFABMS). The infra-red absorptions at 3413 cm^−1^ and 1700–1730 cm^−1^ indicated the presence of hydroxyl and various carbonyl groups. In the ^1^H NMR spectrum, typical peaks associated with limonoid skeleton were identified. The furan side chain at δ 7.64 (*s*), δ 6.29 (*br s*), and δ 7.30 (*d*, J = 1.5 Hz) were attributed to H-21, H-22, and H-23, respectively. Signals arising from oxygenated methine protons of H-3 at δ 4.91 (*d*, J = 10.2 Hz) and H-17 at δ 5.46 (*s*) were easily differentiated and assigned based on the signal multiplicity. On the other hand, the occurrence of an oxygen bridge that links C-1 to C-29 was deemed rare [11]. The isolated diasterotopic methylene protons of H_2_-29 were detected as a pair of doublets at δ 3.46 and δ 3.88, with a coupling constant of 9.3 Hz. At the allylic alcohol substructure, the olefinic signal was found at δ 5.53 (dd, J = 1.7, 7.1 Hz). The cinnamate side chain at C-3 was confirmed by the presence of five aromatic protons in the region δ 7.10–δ 7.20 and the characteristic doublet signals of H-2′ and H-3′ at δ 6.25 (*d*, J = 16.0 Hz) and δ 7.58 (*d*, J = 15.7 Hz). The relative stereostructure of erythrocarpin E was established through nuclear Overhauser effect spectroscopy (NOESY). The chemical structure of CEB4 is shown in Figure 1.

### CEB4 Inhibits the Proliferation of HSC-4 Cells

**Figure 2:**
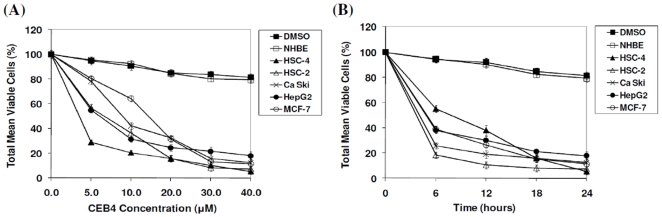
Comparison of total relative cell viability (%) between various human tumor cell lines and normal human bronchial epithelial cells (NHBE) after treatment with CEB4 at different concentrations (0 to 40.0 µM) over 24 h. (A) Dose dependent MTT assay graph upon 24 h of CEB4 exposure. (B) Time dependent MTT assay graph upon treatment with 40.0 µM CEB4. All results were expressed as total percentage of viable cells with mean ±SD of three independent determinations. Solvent controls using DMSO was performed on HSC-4 cells as a representative of all other cell lines.

MTT assays were conducted to assess the cytotoxic attributes and inhibitory concentration (IC_50_) values of CEB4 on five human tumor and NHBE normal cell lines. The results indicated that CEB4 induces cytotoxicity in a dose and time dependent manner over a 40.0 µM treatment regime and 24 h of exposure (Fig. 2A and 2B). Minimal cytotoxic effects were observed on NHBE cells, where approximately 20.0% killing was observed under similar treatment conditions, as opposed to 95% killing in HSC-4 cells with the lowest IC_50_ value recorded of 4.0±1.9 µM among all cell lines tested (Table 1). Viability of cells treated with DMSO without CEB4 were insignificantly affected (<1.0 %) (Fig. 2A and 2B) thereby ruling out the involvement of solvent-induced cytotoxicity. Both time and dose dependent assays supported the need to further investigate the apoptotic effects of CEB4 and its potential as an anti-tumor drug for the treatment of oral cancer. All of the following experiments were carried out based on IC_50_ values as obtained from our present MTT data, and are summarized in Table 1.

**Table 1:** Summary of IC_50_ values and percentage cell viability of CEB4 treated tumor cell lines and NHBE cells as obtained from MTT cell viability assays after 24 h of exposure.

Cell Lines	IC_50_ (µM) †	Cell Viability (%) ††
Normal human bronchial epithelial cells (NHBE)	*n/a*	80.1±3.4
Human oral squamous cell carcinoma (HSC-4)	4.0±1.9	5.2±2.5
Human oral squamous cell carcinoma (HSC-2)	7.0±3.2	7.2±1.9
Human cervical carcinoma (Ca Ski)	8.5±2.8	12.4±3.1
Human hepatocyte carcinoma (HepG2)	6.0±2.5	17.9±3.2
Human breast adenocarcinoma (MCF-7)	14.0±2.4	11.4±3.1

† IC_50_ values at 24 h exposure.

†† Viability upon 24 h exposure with 40 µM CEB4.

All results were expressed as total percentage of viable cells with mean ±SD of three independent determinations.

### CEB4 Induces Apoptosis Mediated Cell Death in HSC-4 Cells

**Figure 3:**
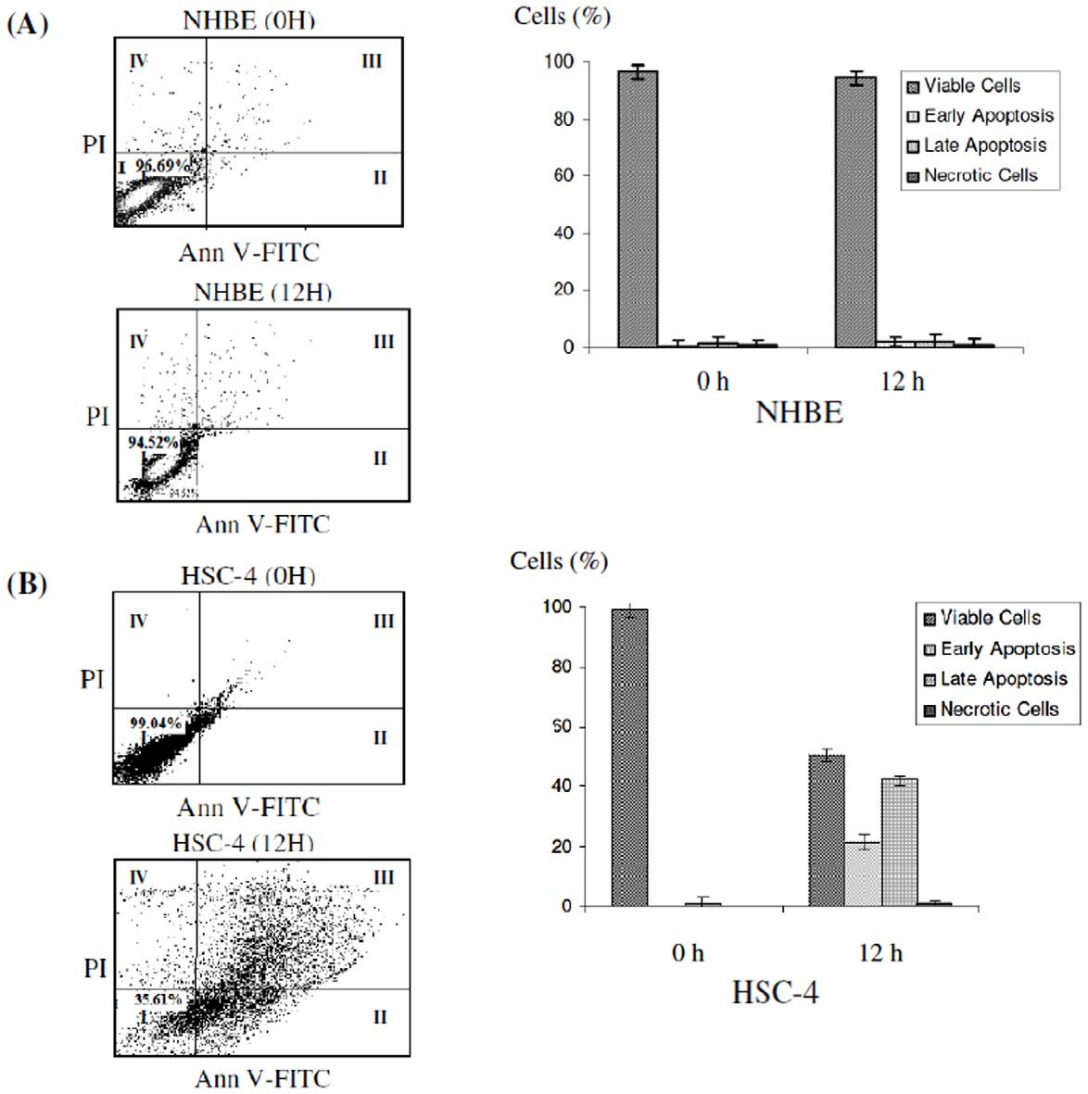
Detection of apoptosis using annexin V-FITC and PI dual staining on (A) NHBE cells and (B) HSC-4 cells. Untreated cells (upper panel) and treated cells (lower panel) after CEB4 treatment at IC_50_ concentrations for 12 h. Quadrants were designed as follows, I: non-stained cells indicating viable cells; II: annexin V-FITC stained cells indicating early apoptosis; III: annexin V-FITC and PI stained cells indicating late apoptosis; and IV: PI stained cells indicating secondary necrosis. All dot plots are a representation of equal cell populations (n = 10,000).

We next determined whether the CEB4 cytotoxic effects were mediated through apoptosis. An increase in cellular staining with FITC-conjugated annexin-V serves as an early marker for apoptosis. Cells were simultaneously stained with PI to investigate loss of cell membrane integrity. This double staining procedure distinguishes early stage apoptotic cells (annexin V-positive) from late stage apoptotic cells (annexin V-positive, PI positive). Treatment of HSC-4 cells at IC_50_ concentrations of CEB4 was found to induce apoptotic cell death through the observation of a shift in viable cell population from early to late stage of apoptosis, followed by secondary necrosis (Fig. 3B). The percentage of viable cells decreased from 99% to 51%, while early and late stage apoptotic cells increased to 21% and 45% respectively. The total percentage of apoptotic HSC-4 cells was 63% after 12 h. No population shifts were observed in the NHBE cells after similar CEB4 treatment (Fig. 3A).

### CEB4 Induces Endonuclease- and Caspase-Mediated Cleavage of DNA and PARP Respectively

**Figure 4:**
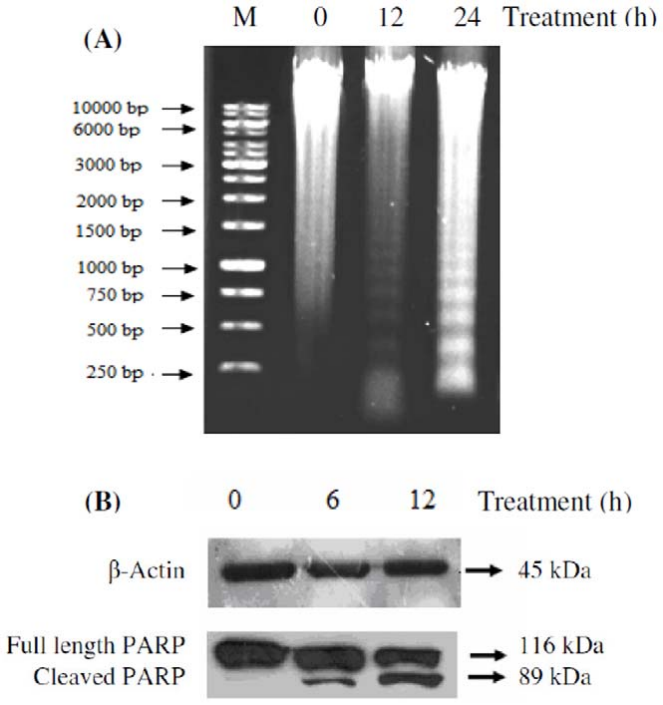
Confirmation of apoptosis mediated cell death in HSC-4 cells through observation of (A) DNA laddering using DNA fragmentation assay on cells treated with CEB4 for 12 and 24 h followed by analysis of extracted DNA on 0.1% (w/v) agarose gel electrophoresis. Smearing of extracted DNA in lane 1 (0 h) was due to the extraction process. However, no distinct fragmentations were observed in lane 1 indicating the absence of apoptosis as opposed to lanes 2 (12 h) and 3 (24 h). (**B**) PARP degradation was observed at different CEB4 incubation periods. Equal amounts of cellular proteins were subjected to SDS-PAGE, and PARP degradation from its native form (116 kDa) to the cleaved form (89 kDa) was detected by Western blot analysis.

Due to the diverse manner and hallmarks in which the process of apoptosis can be manifested, we also examined the induction of apoptosis in HSC-4 cells through the occurrence of DNA fragmentation and cleavage of PARP proteins. We observed that DNA extracted from untreated HSC-4 cells showed no fragmentation, while DNA from CEB4-treated HSC-4 cells (12 and 24 h) showed DNA laddering with approximately 200 bp intervals presumably as the result of endonuclease action at sites between nucleosomes (Fig. 4A). The suspected involvement of caspase-3 activation in CEB4-induced apoptosis was also validated through the expression and degradation of a nuclear protein, PARP. Proteolytic cleavage of full length PARP from a 116-kDa polypeptide to an 85-kDa fragment is a typical marker for the onset of apoptosis, and was observed in a time dependant manner in HSC-4 cells treated with CEB4 (Fig. 4B). Therefore, the apoptosis-inducing effects of CEB4 on HSC-4 cells was confirmed.

### Expression of p53 and other p53-Related Apoptotic Proteins

**Figure 5:**
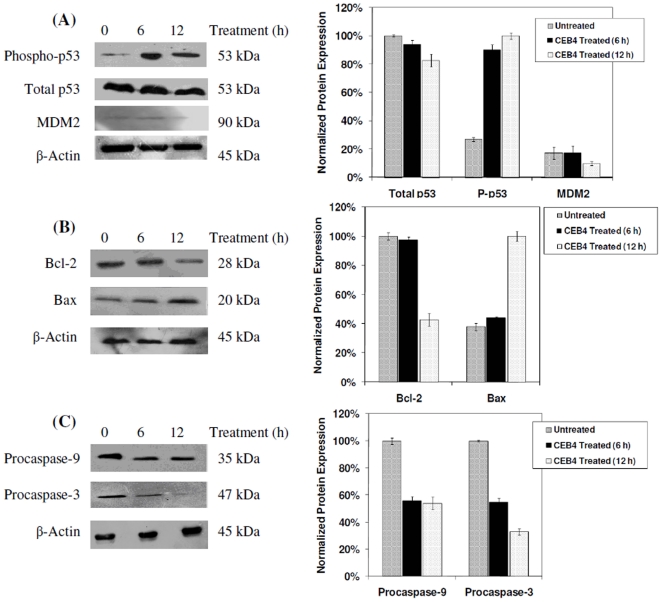
Modulation of p53 signalling and p53-related apoptotic protein levels by CEB4 on HSC-4 oral cancer cells. Cells were incubated with CEB4 for 6 and 12 h, harvested in lysis buffer and subjected to SDS-PAGE. Protein expression levels were examined by Western blot analysis and quantified using the ImageJ software employing β-actin as a normalization control. (**A**) Analysis of p53 and MDM2 inhibitor levels. (**B**) Analysis of anti-apoptotic Bcl-2 and pro-apoptotic Bax protein levels. (**C**) Analysis of initiator procaspase-9 and effector caspase-3 zymogen levels. Quantification of normalized protein levels against β-actin are shown on right panels.

Western blotting analysis of CEB4 treated HSC-4 cells was carried out to examine the status of p53 and p53-related apoptotic proteins. The results showed that upon CEB4 treatment, phosphorylated p53 was elevated, while the level of the p53 inhibitor, MDM2, declined over 12 h (Fig. 5A). The total p53 level was consistent in HSC-4 cells and remained unchanged upon CEB4 exposure. Protein expression levels of the pro-apoptotic Bax protein increased after 12 h of CEB4 treatment. On the other hand, the level of anti-apoptotic Bcl-2 protein was found to decrease concomitantly with the change in Bax (Fig. 5B). We also investigated downstream caspases, and found that the amount of initiator procaspase-9 and executioner caspase-3 zymogen decreased concurrently upon CEB4 exposure, indicating the activated cleavage of procaspase-9 and the caspase-3 zymogen (Fig. 5C).

## Discussion

The process of apoptosis has become the desired route for cancerous cells to die, and is normally characterized by morphological and biochemical changes such as membrane blebbing, cellular shrinkage, chromatin condensation, activation of cascades of proteases such as caspases and endonucleases, cleavage of PARP [12] and fragmentation of genomic DNA [13]. Apoptotic cell death is favoured because it does not involve the sudden release of pro-inflammatory mediators, as in the process of necrosis [14]. The induction of apoptosis and inhibition of cancer cell proliferation have been used as benchmarks in the evaluation of phytochemical anti-cancer activities, where in the past many chemotherapeutic agents have been found to effect disturbance in cell cycle progression or the induction of apoptosis in tumor cells [15].

In this study, evidence from the MTT-based cell viability tests showed that erythrocarpine E (CEB4) induces both time and dose dependent cytotoxic effects on all human tumor cell lines tested, with efficacy being highest in HSC-4 cells. This observation invites further investigation of the apoptotic effects of CEB4 and its potential as an anti-tumor drug for the treatment of oral cancers. At this stage, the epithelial-like origin of NHBE which holds similarities to HSC-4 merely serves as an initial *in vitro* control for drug screening purposes, while the actual physiological side effects of CEB4 can only be evaluated through *in vivo* studies. The minimal cytotoxic effects of CEB4 on NHBE cells (where cell viability levels remained above 80% upon treatment) provide a preliminary safety indication for its further development as a chemotherapeutic agent. Three types of assays were also conducted to confirm the cell death mechanism of CEB4 on HSC-4 cells: annexin V-FITC, PARP cleavage and DNA fragmentation assays. The combined results of these tests indicate that cytotoxicity induced by CEB4 results from the induction of apoptosis.

The next pertinent question would be to determine how is CEB4-dependent apoptosis mediated at the molecular level? Two major routes of programmed cell death can be distinguished: the extrinsic and intrinsic pathways. The action of p53 is indirectly related to the intrinsic cell death pathway where it stimulates a wide network of signals which acts in part through the Bcl-2 family signalling cascade [16]. Previous reports have indicated that mutations in p53 play a vital role in cancer initiation and are very prominent in its linkage to cancers such as colon, lung, oesophagus, breast, liver, brain, reticuloendothelial tissues and hematopoietic tissues [17].

The Western blotting results demonstrate an increase in the level of phosphorylated p53 and a reduction of its inhibitor, MDM2 suggesting that the mode of induction of apoptosis by CEB4 is mediated through the intrinsic pathway. This is further reinforced by observations on mitochondrial proteins, where an increase of pro-apoptotic protein levels, Bax and reduction of anti-apoptotic protein levels, Bcl-2 was seen. The ratio between Bcl-2 and Bax regulated the induction of apoptosis by dimerizing with each other, thereby triggering the release of cytochrome c from the mitochondria [18]-[19]. In addition to CEB4, other natural compounds that have displayed effects on the induction of p53-mediated apoptosis were curcumin, resveratrol, anthocyanins and capsaicin [20]. Previous studies on limonoid-based compounds from *Citrus aurantifolia* have also been shown to induce apoptosis through p53 inactivation [10], [20].

The mechanism of action of cytotoxic drugs does not solely include features such as cell shrinkage and DNA fragmentation, but also activation of p53 signalling molecules leading to the activation of a cascade of caspase action [21]. Caspase cascades can be triggered through two major branches, either through cell surface TNF-superfamily death receptors and the pivotal adaptor protein Fas activated death domain (FADD) leading to activation of caspase-8 and ultimately caspase-3, or via the release of cytochrome c (Cytc) from mitochondria resulting in caspase-9 activity and subsequent caspase-3 action. Caspase-3, represents a convergence point between the two routes, and in turn induces PARP cleavage, chromosomal DNA breaks and finally the breakdown of the cell into apoptotic bodies [22]. Our results suggest that CEB4 exerts its apoptotic effects by acting on caspases-9 and -3, where the level of full length procaspase-9 and caspase-3 zymogen is decreased upon CEB4 exposure, resulting in cleaved, activated forms of caspase-9 (37 kDa and 10 kDa) caspase-3 (17 kDa and 12 kDa). However, apoptotic signal transduction involving death receptor signalling resulting in activated initiator caspase-8, which in turn activates zymogen caspase-3 cannot be ruled out. Mediation of cell death via this route could explain the observation of an early decrease (<6 h) in procaspase-3 levels prior to changes in Bcl-2 and Bax which occur at 12 h (Fig. 5B and 5C). This could also mean that the involvement of the intrinsic pathway serves as an amplification step towards initial death signalling via the extrinsic pathway upon CEB4 exposure.

We conclude that CEB4 induces cytotoxicity through apoptotic cell death in HSC-4 oral cancer cells, and that the induction of apoptosis is brought about by the activation of p53, which triggers the intrinsic pathway through Bcl-2 and Bax, and finally through caspase-9 and -3 activation. Our data indicates the potential for the development of CEB4 as a novel potential anti-cancer drug against human oral cancer, although further work on *in vivo* safety effects and pharmacokinetics is required before CEB4 can be fully evaluated in a clinical setting.
