# Correction: Regulation of Apoptotic Effects by Erythrocarpine E, a Cytotoxic Limonoid from *Chisocheton erythrocarpus* in HSC-4 Human Oral Cancer Cells

**DOI:** 10.1371/annotation/2f4528dd-653e-4fa2-a381-aa804e47ec7a

**Published:** 2011-09-02

**Authors:** Noor Hasima Nagoor, Norliza Shah Jehan Muttiah, Chong Soon Lim, Lionel L. A. In, Khalit Mohamad, Khalijah Awang

The 5th author's last name is misspelled. The correct 5th author's name is: Khalit Mohamad.

The correct citation is: Nagoor NH, Shah Jehan Muttiah N, Soon Lim C, In LLA, Mohamad K, et al. (2011) Regulation of Apoptotic Effects by Erythrocarpine E, a Cytotoxic Limonoid from Chisocheton erythrocarpus in HSC-4 Human Oral Cancer Cells. PLoS ONE 6(8): e23661. doi:10.1371/journal.pone.0023661 

